# Anti-Aging Effects of *Leontopodium alpinum* (Edelweiss) Callus Culture Extract through Transcriptome Profiling

**DOI:** 10.3390/genes11020230

**Published:** 2020-02-21

**Authors:** Won Kyong Cho, Hye-In Kim, Soo-Yun Kim, Hyo Hyun Seo, Jihyeok Song, Jiyeon Kim, Dong Sun Shin, Yeonhwa Jo, Hoseong Choi, Jeong Hun Lee, Sang Hyun Moh

**Affiliations:** 1 Research Institute of Agriculture and Life Sciences, College of Agriculture and Life Sciences, Seoul National University, Seoul 08826, Koreayeonhwajo@gmail.com (Y.J.); 2 Anti-Aging Research Institute of BIO-FD&C Co., Ltd., Incheon 21990, Korea; hikim@biofdnc.com (H.-I.K.); sykim@biofdnc.com (S.-Y.K.); hhseo@biofdnc.com (H.H.S.); jhsong@biofdnc.com (J.S.); jykim@biofdnc.com (J.K.); dsshin@biofdnc.com (D.S.S.); jhlee@biofdnc.com (J.H.L.); 3 Department of Agricultural Biotechnology, College of Agriculture and Life Sciences, Seoul National University, Seoul 08826, Korea; bioplanths@gmail.com

**Keywords:** anti-aging, callus, edelweiss, skin cells, transcriptome

## Abstract

Edelweiss (*Leontopodium Alpinum*) in the family *Asteraceae* is a wildflower that grows in rocky limestone places. Here, we investigated the efficacy of edelweiss callus culture extract (*Leontopodium Alpinum* callus culture extract; LACCE) using multiple assays from in vitro to in vivo as well as transcriptome profiling. Several in vitro assay results showed the strong antioxidant activity of LACCE in response to UVB treatment. Moreover, LACCE suppressed inflammation and wrinkling; however, moisturizing activity was increased by LACCE. The clinical test in vivo demonstrated that constant application of LACCE on the face and skin tissues improved anti-periorbital wrinkles, skin elasticity, dermal density, and skin thickness compared with the placebo. The RNA-Sequencing results showed at least 16.56% of human genes were expressed in keratinocyte cells. LACCE up-regulated genes encoding several KRT proteins; DDIT4, BNIP3, and IGFBP3 were involved in the positive regulation of the developmental process, programmed cell death, keratinization, and cornification forming skin barriers, which provide many advantages in the human skin. By contrast, down-regulated genes were stress-responsive genes, including metal, oxidation, wounding, hypoxia, and virus infection, suggesting LACCE did not cause any harmful stress on the skin. Our comprehensive study demonstrated LACCE is a promising agent for anti-aging cosmetics.

## 1. Introduction

Edelweiss (*Leontopodium nivale* subsp. *Alpinum* (Cass.) Greuter) in the family *Asteraceae* is a wildflower that grows in rocky limestone places at high altitude, such as the Swiss Alps [1,2]. Due to the rarity of its short-lived white flowers, edelweiss represents beauty and purity related to the Alps and Carpathians. In addition, many countries, including Austria, Bulgaria, Romania, Slovenia, and Switzerland, regard edelweiss as a national symbol. 

For a long time, edelweiss has been used as traditional medicine against abdominal aches, bronchitis, diarrhea, dysentery, and fever [3,4]. Recently, several studies have shown the efficacy of edelweiss extracts for anti-inflammation in mice and rats [3] and human keratinocytes and endothelial cells [4]. In addition, the root extracts of edelweiss contain constituents that enhance cholinergic neurotransmission, indicating its potential for antidementia agents [5] and antioxidants, such as leontopodic acid A and 3,5-dicaffeoylquinic acid, which can be used as anti-aging agents [6]. Moreover, the edelweiss extracts showed antibacterial activity against *Enterococcus faecium*, *Escherichia coli*, *Pseudomonas aeruginosa*, *Staphylococcus aureus*, *Streptococcus pneumoniae*, and *Streptococcus pyogenes*, suggesting their possible ethnomedicinal use for respiratory and abdominal disorders [7].

Several previous studies have analyzed the compounds of edelweiss extracts. For instance, the capillary chromatography method revealed 12 pharmacologically-important compounds, including flavonoids, caffeic acids, and leontopodic acid, from the aerial parts of edelweiss [8]. To extract antioxidants from edelweiss plants, centrifugal partition chromatography (CPC) and high-performance liquid chromatography (HPLC) methods have been developed [6]. It is known that the active compounds of edelweiss extracts between the aerial parts and roots are diverse [3]. For instance, the hairy roots of edelweiss produce pharmacologically-active lignans, such as leoligin and 5-methoxy-leoligin, which can be stimulated by several other components, such as silver nitrate, sucrose, methyl jasmonate, and yeast extract [9]. Furthermore, the metabolic patterns of 11 different *Leontopodium* species have been revealed by nuclear magnetic resonance (1H NMR) spectroscopy and liquid chromatography–mass spectrometry (LC–MS) in their taxonomy relationship [10]. 

Plant callus can be defined as unorganized or undifferentiated cell masses, which are easily induced by wounding to cover a plant wound or artificially conducting an in vitro system. Plant callus is artificially cultured by adding nutrients and plant growth regulators in antiseptic growth conditions. Currently, it is possible to produce a high number of specific plant cells with equal quality in a bioreactor. Moreover, a plant cell has an ability referred to as totipotency, which is the genetic potential of a plant cell to produce the entire mature plant [11]. Thus, it is possible to generate a mature plant from the specific plant callus. Furthermore, callus can be widely used for plant regeneration to conserve rare and endangered plants [12,13]. 

Like animal cells, plant cells have abilities to facilitate the stimulation and regeneration of plants after injury [14]. Although plant stem cells are regarded as emerging materials in the cosmetic industry, the available materials are limited. It is important to identify new plant sources and evaluate their functional components associated with cosmetics. 

Edelweiss extracts are well known for their use in pharmaceutical agents; however, the effects of edelweiss extract as a natural cosmetic source are rarely reported. Here, we investigated the efficacy of edelweiss callus extract (*Leontopodium Alpinum* callus culture extract; LACCE) derived from edelweiss leaves using multiple assays from in vitro to in vivo and revealed the molecular mechanism caused by LACCE using transcriptome profiling. 

## 2. Materials and Methods 

### 2.1. Production of Edelweiss Callus 

Edelweiss seeds were commercially purchased. The edelweiss seeds were soaked in 70% ethanol for 30 s followed by washing with distilled water. Again, the seeds were shaken in 0.3% sodium hypochlorite (Waco, Osaka, Japan) for 20 min and washed with distilled water. The sterilized seeds were germinated on basic Murashige and Skoog (MS) medium (Duchefa Biochemie, Haarlem, The Netherlands). The edelweiss leaves in the aseptic condition were cut into small pieces (0.5 to 1 cm). We induced the early stage of plant cells on MS medium containing 0.5–3 mg/mL of 6-Benzylaminopurine (6-BAP) (Duchefa Biochemie) and 0.3–1 mg/mL of 2,4-Dichlorophenoxyacetic acid (2,4-D) (Duchefa Biochemie) in darkness at 25 ± 2 °C [15]. The pH of the MS medium was adjusted to 5.8 using 1N of NaOH (Duchefa Biochemie). The induced callus was propagated in the petri dish. The selected callus line was cultured in a bio-reactor in the Anti-Aging Research Institute of BIO-FD&C Co., Ltd., Incheon, Korea. The cultured callus was harvested and washed three times with distilled water. The callus was dehydrated using the freeze drier (IlShinbioBase, Dongducheonsi, Korea) according to the manufacturer’s instructions. The dried edelweiss callus was stirred in distilled water at 50 °C for 8 h. Callus extracts were obtained by heat extraction at 98 °C for 10 min.

### 2.2. Culture of Human Skin Cells 

The evaluated effects of edelweiss extracts on human skin cells, keratinocyte (HaCaT) cells, and normal human Detroit 551 fibroblast (ATCC, Manassas, VA, USA) were cultivated in Dulbecco’s Modified Eagle Medium (DMEM) (Welgene, Gyeongsan-si, Korea) supplemented with 10% fetal bovine serum (FBS) (Thermo Fisher Scientific, Waltham, MA, USA) and 1% antibiotic-antimycotic (Thermo Fisher Scientific, Waltham, MA, USA) at 37 °C with a 5% CO_2_ condition. 

### 2.3. Assessment of Cell Metabolic Activity by MTT Assay

To assess the cytotoxicity of LACCE on the cellular growth, propagation, and survival of human skin cells, an MTT (3-(4,5-dimethylthiazol-2-yl)-2,5-diphenyltetrazolium bromide) assay was carried out as described previously [16,17]. In brief, HaCaT and Detroit 551 cells at a density of 5 × 10^4^ cells per well, respectively, were incubated in a 96-well plate for 24 h. After that, final concentrations of 0.1%, 0.5%, and 1% LACCE were treated for 24 h. Distilled water was used as a control. After the treatment of LACCE, the medium was removed followed by the addition of 4 µL of 5 mg/mL MTT (Sigma-Aldrich, St. Louis, MO, USA) and incubated for 4 h. After removing the medium, 100 µL of dimethylsulfoxide (DMSO) (Sigma) was added and dissolved for 10 min. The wavelength absorbance was measured at 570 nm using a Thermo Scientific Multiskan GO Microplate Spectrophotometer (Fisher Scientific Ltd., Vantaa, Finland). Cell viability was obtained using the following formula. Cell viability (%) = (the amount of absorbance for treated cells/the amount of absorbance of control cells) × 100.

### 2.4. Assessment of Antioxidant Activity by DPPH Assay

The antioxidant activity of LACCE was measured by a DPPH (2,2-diphenyl-1-picryl-hydrazyl-hydrate) assay as described previously [18]. In brief, 0.1 mL of final concentrations of 0.1%, 0.5%, and 1% LACCE was treated in 0.1 mL of 0.1 mM of DPPH (Sigma-Aldrich) in the presence of 0.4 mL of ethanol. We used 0.001% ascorbic acid (vitamin C) (Sigma-Aldrich) as a positive control. The samples were mixed well for 10 s and incubated at room temperature in dark conditions for 30 min. The wavelength absorbance was measured at 517 nm using a Thermo Scientific Multiskan GO Microplate Spectrophotometer (Thermo Fisher Scientific, Waltham, MA, USA). The radical scavenging activity was calculated using the following formula. Scavenging activity (%) = [1-(absorbance of test sample/absorbance of control)] × 100.

### 2.5. Assessment of Antioxidant Activity by Hydrogen Peroxide (H_2_O_2_) Assay

Hydrogen peroxide produces oxygen-free radicals in cells, resulting in cell death. We tested the cell mortality rate caused by hydrogen peroxide in LACCE-treated samples. HaCaT cells at a density of 5 × 10^5^ cells per well were incubated in a 24-well plate for 24 h. After that, final concentrations of 0.1%, 0.5%, and 1% LACCE were treated in the presence of 1 mM of H_2_O_2_ for 8 h. As a positive control, 0.033% NAC (Sigma-Aldrich) was used. An MTT assay was used to measure the cell survival rate of LACCE-treated samples caused by H_2_O_2_. In addition, we observed cell morphology by methylene blue staining (Sigma-Aldrich, St. Louis, MO, USA). 

### 2.6. Real-Time Reverse Transcription (RT)-PCR

To examine the effect of LACCE on anti-wrinkles, moisturizing, and anti-inflammation, we carried out real-time RT-PCR with known primers amplifying marker genes using QuantiTect Primer Assays (Qiagen, Hilden, Germany) according to the manufacturer’s instructions. Detroit 551 cells at a density of 5 × 10^4^ cells per well were incubated in a 96-well plate for 24 h. After that, final concentrations of 0.1%, 0.5%, and 1% LACCE was treated for 24 h. cDNA was synthesized from the respective Detroit 551 cells treated with different LACCE concentrations using a SuperPrep Cell Lysis & RT Kit for qPCR (Toyobo, Osaka, Japan) containing lysis reagents and RT reagents according to the manufacturer’s instructions. For the anti-wrinkle effect upon LACCE treatment, the expression of the gene encoding Matrix Metalloproteinase-2 (MMP-2) was quantified by real-time RT-PCR using a Thunderbird SYBR qPCR Mix kit (Toyobo) based on the manufacturer’s instructions. For the evaluation of moisturizing through LACCE treatment, the expression of the gene encoding Aquaporin 3 (AQP3) was examined by real-time RT-PCR. In the case of the anti-inflammatory assay, HaCaT cells at a density of 5 × 10^4^ cells per well were incubated in a 96-well plate for 24 h. After removing the medium, UVB was irradiated on the HaCaT cells. For UVB irradiation, 5 mJ/cm^2^ was used by CL-1000 UV box (Analytik Jena AG, Jena, Germany). After that, final concentrations of 0.1%, 0.5%, and 1% LACCE were treated for 4 h. We used dexamethasone (Dex) (Sigma-Aldrich) as a positive control. For the anti-inflammatory effect from LACCE treatment, the expression of genes encoding COX2 and iNOS, respectively, were quantified by real-time RT-PCR. The expression of individual genes was normalized to GAPDH gene expression. 

### 2.7. Clinical Evaluation of LACCE as an Agent for Cosmetics in Vivo

Clinical study for the evaluation of the efficacy of LACCE on facial lifting and improving periorbital wrinkle, skin elasticity, dermal density and skin thickness was conducted by Ellead company after the approval based on standard operating procedures (Ver. 3.0) (Ellead, Seongnam, Korea) of Ellead IRB in accordance with Korea Good Clinical Practices guideline (B1-2015-4-002) described by the Ministry of Food and Drug Safety (MFDS) and Ellead Standard Operating Procedures (EL-P-7400).

To examine the effect of LACCE in vivo, we carried out a clinical evaluation of LACCE for four different factors: facial lifting and improving periorbital wrinkles, skin elasticity, dermal density, and skin thickness. For this, a total of 21 female volunteers with average 48.04 ± 4.28 years old, who were further divided into 12 volunteers aged in their 40s and nine volunteers aged in their 50s, participated in the clinical evaluation over four weeks. 

The clinical test was conducted at three different time points, the baseline, 2 weeks, and 4 weeks, for all volunteers. From a week before the beginning of the study until the end, all volunteers did not undergo additional treatments to improve skin, cosmetics, and sanitary aid, which could have affected the results. After washing the faces of the volunteers using the cleansing foam, the volunteers stood for at least 30 min in a controlled room with a constant temperature (20–24 °C) and humidity and (40%–60% relative humidity). We performed the measurement randomly on the left or right side of the faces of volunteers. Our test was a double-blind test in which neither the subjects nor the researchers knew which product was a test sample. Formulations to calculate the decreased and increased rates were described in Table 1. 

### 2.8. Measurement of Facial Lifting and Improving Periorbital Wrinkles

At the baseline, periorbital wrinkles, skin elasticity, dermal density, and skin thickness on the cheek, and facial lifting at the corner of the mouth were measured for all volunteers. After that, two different samples, LACCE and a placebo (control), were applied to the designated area of the face twice a day (morning and night). Volunteers were divided into two different groups, group I (LACCE was applied to the left facial area while the placebo was applied to the right facial area) and group II (the placebo was applied to the left facial area while LACCE was applied to the right facial area). Two and four weeks after treatment, the same measurements were conducted to evaluate the effect of LACCE compared with that of the placebo. 

Periorbital wrinkles were analyzed using the optical 3D (dimension) skin measurement system PRIMOS High Resolution (Canfield Scientific, Parsippany, NJ, USA), which is ideal for the investigation of the skin microstructure and wrinkles. Two different roughness parameters, Ra (average roughness), which is the average of the absolute values of the profile heights of the roughness profile, and Rq (root mean square roughness), which is the root mean square average of the profile heights of the roughness profile, were measured.

### 2.9. Measurement of Skin Elasticity

Skin elasticity was analyzed using a Cutometer MPA 580 (Courage & Khazaka, Köln, Germany). A probe with a diameter of 2 mm was mounted on this equipment for the test. Mode 1 or the time-strain mode was used as the measurement condition. On-time 2.0 s and off-time 2.0 s were then applied to the specified negative pressure of the mode 1 condition (450 mbar), and measurements were taken three consecutive times. Three different parameter values, R2 (gross elasticity), R5 (net elasticity), and R7 (biological elasticity), were measured. 

### 2.10. Measurement of Dermal Density and Skin Thickness

The dermal density and skin thickness on the cheek were analyzed using Dermascan-C (Cortex Technology, Hadsund, Denmark), a high-resolution imaging machine using 20-MHz supersonic waves, which is very useful in non-invasively observing changes to the inner layers of the skin. In our study, the B scanning image was used. First, we set the speed of supersonic waves to 20 MHz, spread the contact jelly for the test, placed the probe at a right angle to the skin, and pressed it slightly to measure the cheek area. The skin thickness and dermal density were calculated by the Dermascan-C software system. 

The images of the face were photographed with identical photography conditions, such as the same light, high-resolution digital camera, and photographer. After that, facial lifting at the corner of the mouth was examined using Moiré analysis based on the images of the face. We selected the corner of the mouth, where skin sagging is prominent, as the test area. Using the images, the angle (R) between the horizontal line and the contour line drawn at the corner of the mouth was calculated using image analysis software (ImagePro Plus, Rockville, MD, USA). 

### 2.11. Statistical Test

We conducted a one-way ANOVA test for the comparison between the control and test samples. The results were shown with mean and standard deviation (Mean ± SEM). The *p*-values *p <* 0.05 (*), *p <* 0.01 (**), and *p <* 0.001 were considered statistically significant. All statistical tests were declared statistically significant at the 0.05 level. We used IBM SPSS Statistics version 21.0 (SPSS, Chicago, IL, USA) for the statistical analysis.

### 2.12. Preparation of Libraries for RNA-Seq and Next-Generation Sequencing

For transcriptome analysis, HaCaT cells at a density of 1 × 10^6^ cells per well were incubated in a six-well plate for 24 h. After that, human keratinocyte cells (treatment) were treated with a final concentration of 1% LACCE extract for 24 h while the mock condition (control) was treated with sterile water. For each condition, three different biological samples were harvested. Total RNA was extracted using an RNeasy Mini Kit (Qiagen, Hilden, Germany) according to the manufacturer’s instructions. After the extraction of total RNAs from each sample, six different libraries for RNA-Seq were prepared by the TruSeq Stranded mRNA LT Sample Prep Kit according to the manufacturer’s instructions. The six different libraries were paired-end sequenced by Illumina’s NovaSeq 6000 system (Macrogen, Seoul, Korea). The obtained raw sequence data were deposited in the National Center for Biotechnology Information (NCBI) Sequence Read Archive (SRA) database with the following respective accession numbers: SRR9127735, SRR9127736, SRR9127737, SRR9127738, SRR9127733, and SRR9127734.

### 2.13. Mapping, Normalization, and Identification of Differentially-Expressed Genes

We mapped the raw sequence reads from each library to human reference transcripts version GRCh38 (https://www.ncbi.nlm.nih.gov/genome/guide/human/) using the BBMap aligner with default parameters (https://sourceforge.net/projects/bbmap/). Using the bbmap.sh option, we calculated fragments per kilobase million (FPKM) values for each transcript. The obtained FPKM values from each condition were subjected to DEBrowser for the normalization and analysis of differentially-expressed genes (DEGs) [19]. FPKM values of less than 1 were deleted. Using EdgeR with the TMM normalization method and exactTest type, we identified differentially-expressed genes according to adjusted *p*-values of less than 0.001 and log_2_ converted fold changes of more than one. 

### 2.14. Gene Ontology (GO) Term Enrichment Analysis

For GO term enrichment analysis, we identified DEGs based on adjusted *p*-values of less than 0.05 and log2 converted fold changes of more than 0.5. As a result, 22 up-regulated and 13 down-regulated genes were identified. Each gene set was subjected to GO enrichment analysis using the GORILLA program with default parameters (http://cbl-gorilla.cs.technion.ac.il/) [20]. We selected two unranked lists of genes as the running mode using 11,290 expressed genes as the background set.

## 3. Results

### 3.1. Preparation of LACCE from Edelweiss

**Figure 1:**
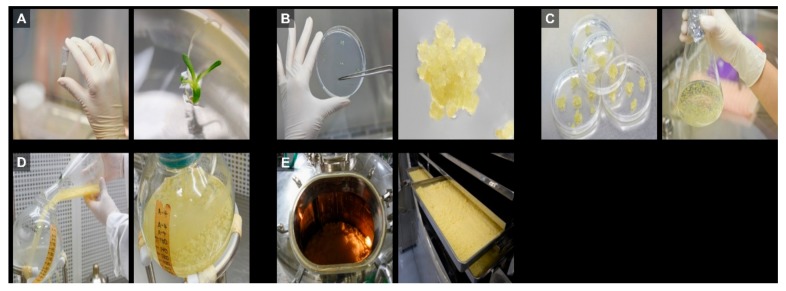
Experimental procedure to obtain *Leontopodium Alpinum* callus culture extract (LACCE) from edelweiss callus using a bioreactor. (**A**) Edelweiss seeds were sterilized and germinated. (**B**) Callus was induced from edelweiss leaf tissue. (**C**) Induced callus was suspension cultured. (**D**) Obtained cells were cultured in a bioreactor. (**E**) Callus cells were harvested and lyophilized in a large quantity.

LACCE was obtained as shown in Figure 1. In brief, edelweiss seeds were first sterilized and germinated (Figure 1A). Callus was induced from the leaves of germinated seeds (Figure 1B) and subjected to the suspension cell culture (Figure 1C). Suspensions cells were cultured in bioreactors for the large-scale production of LACCE (Figure 1D). Cultured cells were harvested and lyophilized for further study (Figure 1E). LACCE was prepared by heat extraction. The different LACCE concentrations were used for multiple assays in vitro (0.1%, 0.5%, and 1%), in vivo (1%), and transcriptome (1%) analyses. 

### 3.2. In Vitro Evaluation of LACCE as an Anti-Aging Agent

**Figure 2:**
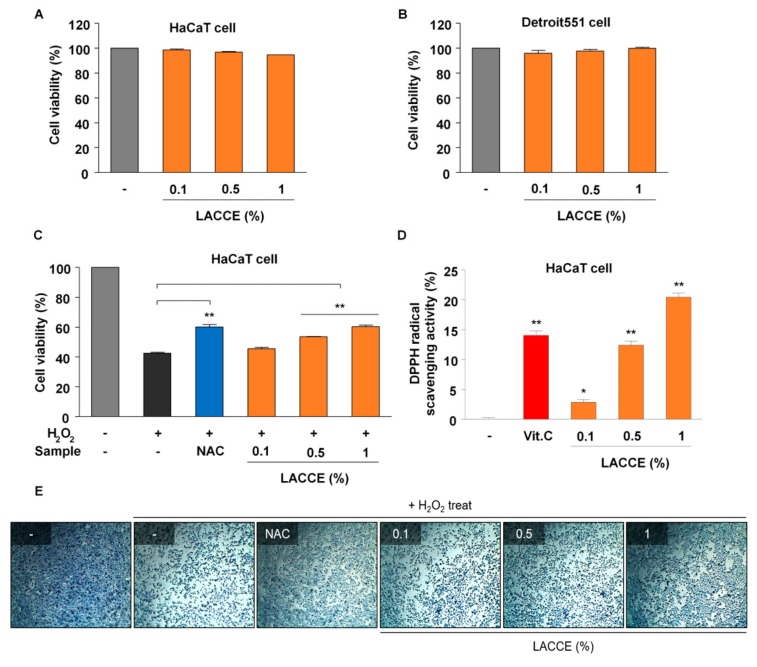
In vitro assessment of LACCE as an anti-aging agent, including cytotoxicity and anti-oxidant activity. Cell viability for three different concentrations (0.1%, 0.5%, and 1%) of LACCE in HaCaT cells (**A**) and Detroit551 cells (**B**) by MTT assay. Gray bar indicates cells treated with distilled water. (**C**) HaCaT cells were treated with hydrogen peroxide. Cell viability for three different LACCE concentrations was measured by 3-(4,5-dimethylthiazol-2-yl)-2,5-diphenyl tetrazolium bromide (MTT) assay. N-acetyl cysteine (NAC) was used as a positive control. (**D**) Antioxidant activity of different LACCE concentrations was measured by DPPH assay. Vitamin C (Vit. C) was used as a positive control. * and ** indicate statistical significance with *p*-values of less than 0.05 and 0.01, respectively. (**E**) Cell morphology in each sample treated with hydrogen peroxide was visualized with methylene blue staining.

We carried out 3-(4,5-dimethylthiazol-2-yl)-2,5-diphenyl tetrazolium bromide (MTT) assay to observe cell viability after treatment with LACCE. In this study, we used immortalized cells instead of primary human skin cells for following reasons. Immortal cells lines have many benefits including being easy to work with, inexpensive, and the unlimited supply of material. In addition, we can skip many ethical problems related to the use of human tissues. Furthermore, immortalized cell lines are preferentially used for the study of basic biological processes such as RNA-Sequencing (RNA-Seq). Two different cell lines, HaCaT and Detroit551, were treated with three different LACCE concentrations (0.1%, 0.5%, and 1%). The cell viability of both HaCaT and Detroit551 cells after treatment with LACCE was comparable to that of the control (Figure 2). In detail, the cell viability of HaCaT cells was slightly reduced from 98.61% (0.1% LACCE) to 94.72% (1% LACCE) as the concentration of LACCE was increased (Figure 2A). By contrast, the cell viability of Detroit551 cells was slightly increased from 95.99% (0.1% LACCE) to 99.95% (1% LACCE) as the concentration of LACCE was increased (Figure 2B).

In addition, we conducted a hydrogen peroxide (H_2_O_2_)-induced MTT assay. In general, hydrogen peroxide caused strong cytotoxicity (42.52% of cell viability) compared with the negative control, whereas the addition of N-acetyl cysteine (NAC), an antioxidant in the cell, inhibited cytotoxicity caused by hydrogen peroxide (60.24% of cell viability). As the concentration of LACCE was increased, the cell viability was increased from 45.54% (0.1% LACCE) to 60.37% (1% LACCE) in response to hydrogen peroxide (Figure 2C). In particular, the reactive oxygen species (ROS) inhibition rate for the 1% LACCE extract was comparable to that of NAC. Moreover, cell morphology was also observed after staining with methylene blue, showing the increase in cell viability from LACCE (Figure 2E). 

Next, we performed an antioxidant assay using a 2,2-Diphenyl-1-picrylhydrazyl (DPPH) radical scavenging assay (Figure 2D). The DPPH radical-scavenging activity was increased from 2.85% (0.1% LACCE) to 20.47% (1% LACCE) as the concentration of the LACCE extract was increased. Compared with vitamin C (14.05%), both the 0.1% and 0.5% LACCE concentrations showed lower radical-scavenging activity while the 1% concentration of the LACCE extract displayed higher radical-scavenging activity. 

### 3.3. In Vitro Evaluation of LACCE Extract as an Anti-Inflammatory Agent

**Figure 3:**
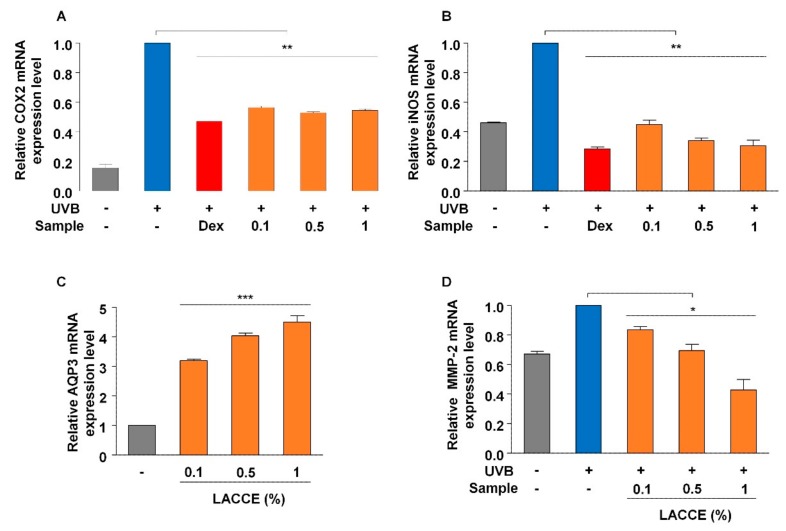
In vitro assessment of LACCE as anti-inflammatory, moisturizing, and anti-wrinkle agents by real-time RT-PCR. Relative expression of COX2 (**A**) and iNOS (**B**), two inflammatory marker genes, in different LACCE concentrations in response to UVB-treated samples was measured by real-time RT-PCR. Dexamethasone (Dex) was used as a positive control. Gray and blue bars indicate HaCaT cells without and with UVB treatment, respectively. (**C**) Relative expression of AQP3, a positive marker for moisturizing effect, in different LACCE concentrations. (**D**) Relative expression of MMP-2, a cell growth marker, in different LACCE concentrations in response to UVB. Expression of individual gene was normalized to GAPDH gene expression. * and ** indicate statistical significance with *p*-values of less than 0.05 and 0.01, respectively.

To examine the anti-inflammatory effect of LACCE, the expression of two inflammatory marker genes encoding COX-2 and inducible nitric oxide synthase (iNOS), respectively, were quantified with real-time RT-PCR (Figure 3A,B). In general, UVB irradiation up-regulated the expression of the COX-2 gene encoding a protein that induces inflammation (Figure 3A). Compared with the negative control, the addition of LACCE to the HaCaT cell significantly suppressed the expression of the COX-2 gene. However, there were no significant differences among the different LACCE concentrations. Compared with the positive control containing dexamethasone (Dex), the LACCE (0.1% to 1%) showed a very similar anti-inflammatory effect. The expression of the iNOS gene was inhibited by the positive control containing Dex and LACCE (Figure 3B). As the concentration of the LACCE extract was increased, the expression of the iNOS gene was gradually reduced. 

### 3.4. In Vitro Evaluation of LACCE Extract Associated with the Moisturizing Effect

We examined the moisturizing effect of LACCE on HaCaT cells using the expression of Aquaporin 3 (AQP3), which encodes a water and glycerol-transporting protein expressed in skin cells, by real-time RT-PCR (Figure 3C). Compared with the negative control, the AQP3 gene was significantly up-regulated by the addition of the LACCE extract. Interestingly, as the concentration of the LACCE extract was increased, the expression of AQP3 was gradually increased from 3.19 (0.1% LACCE) to 4.5 (1% LACCE). 

### 3.5. In Vitro Evaluation of LACCE Extract as an Anti-Wrinkle Agent

We tested the anti-wrinkle effect of LACCE using a marker gene encoding matrix metalloproteinase-2 (MMP-2), which promotes cell growth, by real-time RT-PCR (Figure 3D). UVB irradiation significantly increased the expression of MMP-2 compared with the negative control without any UVB irradiation (fold change 0.67 with *p*-value less than 0.05). The expression of MMP-2 was down-regulated by the addition of LACCE against UVB irradiation. In general, the expression of MMP-2 was gradually decreased as the concentration of LACCE was increased. The expression of MMP-2 in normal conditions without any UVB irradiation was comparable to that in 0.5% LACCE with UVB irradiation. 

### 3.6. Clinical Evaluation of LACCE on Facial Lifting and Improving Periorbital Wrinkles, Skin Elasticity, Dermal Density, and Skin Thickness

To examine the effect of LACCE in vivo, we carried out a clinical evaluation of LACCE associated with facial lifting and improving periorbital wrinkles, skin elasticity, dermal density, and skin thickness. For this, 21 female volunteers, who were further divided into 12 in their 40s and nine in their 50s, participated in the clinical evaluation over four weeks.

**Figure 4:**
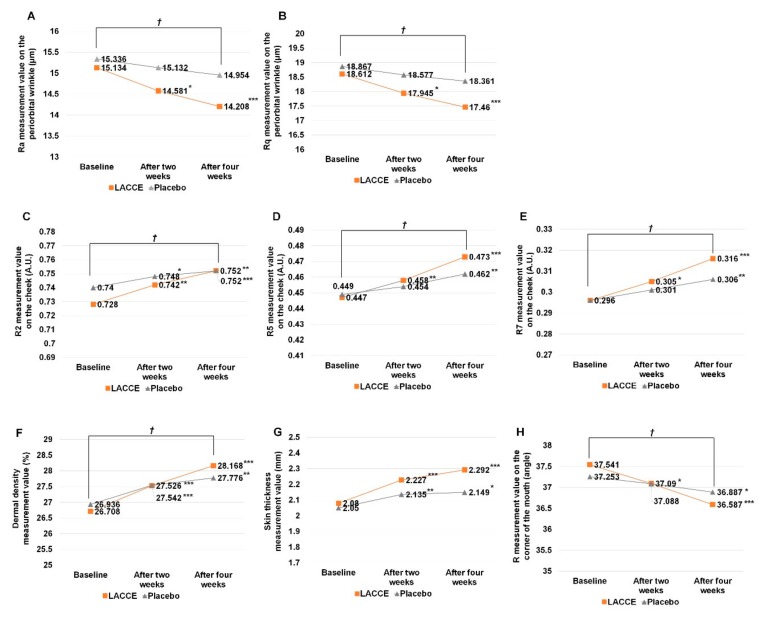
In vivo clinical tests of LACCE for facial lifting and improving periorbital wrinkles, skin elasticity, dermal density, and skin thickness. Ra (**A**) and Rq (**B**) values were measured by the PRIMOS high-resolution system at three different time points to observe periorbital wrinkle effect of LACCE (1%). Gross elasticity (R2) (**C**), net elasticity (R5) (**D**), and biological elasticity (R7) (**E**) values were measured to observe skin elasticity with LACCE. Measurement of dermal density (**F**) and skin thickness (**G**). R measurement at corner of mouth (**H**). Before/after: probability p (repeated measures ANOVA, significant: * *p <* 0.05, ** *p <* 0.01, *** *p <* 0.001). LACCE/Placebo B: probability *p* (repeated measures ANOVA, significant: † *p <* 0.05).

First, we examined the effect of LACCE on periorbital wrinkles using the PRIMOS high- resolution system. Two different roughness parameters, roughness average (Ra) (Figure 4A) and root mean square roughness (Rq) (Figure 4B), were analyzed. Four weeks after treatment with LACCE and the placebo, the Ra and Rq values decreased in both samples. However, the rate of decrease for the Ra and Rq values was much higher in the LACCE-treated sample than in the placebo-treated sample (Table 2). For example, the decrease rate for the Ra value was 3.654% and 6.118% at two and four weeks, respectively, with statistical significance (*p <* 0.05). Similarly, the decrease rate for the Rq value was 3.583% and 6.189% at two and four weeks, respectively, with statistical significance (*p <* 0.05). Moreover, the ratio of volunteers who displayed a reduction in the roughness parameters for the LACCE-treated sample was much higher than that for the placebo-treated sample. For example, at four weeks, the ratios of volunteers showing a decrease in Ra and Rq values were 90.476% in the LACCE-treated sample and 57.142% in the placebo-treated sample. 

We examined the effect of LACCE on cheek skin elasticity using Cutometer. Three different parameters, gross elasticity (R2), net elasticity (R5), and biological elasticity (R2) (R7), were measured (Figure 4C–E). The R2, R5, and R7 values for both the LACCE- and placebo-treated condition increased during the four weeks. For instance, the R2 value for the LACCE-treated sample increased from 0.728 to 0.752, while the R2 value for the placebo-treated sample increased from 0.740 to 0.752 (Figure 4C). Moreover, the increase rate in the LACCE-treated sample was much higher than that in the placebo-treated sample (Table 3). For instance, the increase rates in the LACCE-treated sample were 3.296 (R2), 5.816 (R5), and 6.756 (R7), while the increase rates in the placebo-treated sample were 1.621 (R2), 2.895 (R5), and 3.378 (R7) at four weeks. However, there was no difference in the ratio of volunteers showing an increase in skin elasticity between the two samples. 

We examined the effect of LACCE on dermal density and skin thickness using Dermascan-C. Both samples showed an increase in dermal density and skin thickness four weeks after treatment (Figure 4F,G). For example, the dermal density in the LACCE-treated sample increased from 26.708 to 28.168, whereas that of the placebo-treated sample increased from 26.936 to 28.168 (Figure 4F). The increase rate for dermal density was much higher in the LACCE sample (5.466%) than in the placebo-treated sample (3.118%) four weeks after treatment (Table 4). Again, the increase rate for skin thickness was much higher in the LACCE sample (10.192%) than in the placebo-treated sample (4.829%) four weeks after treatment. Interestingly, many volunteers showed a strong increase in dermal density (90.476%) and skin thickness (100%) after four weeks of LACCE treatment. 

We examined the effect of LACCE on facial lifting at the corner of the mouth using Moiré analysis. Both the LACCE- and placebo-treated samples showed a reduction in facial lifting (Figure 4H); however, the decrease rate of facial lifting was much higher in the LACCE sample (2.541%) than that in the placebo sample (0.437%) at four weeks of treatment. The ratio of volunteers showing a decrease in facial lifting was higher with LACCE (85.714%) than the placebo (57.142%).

### 3.7. Transcriptome Analysis of HaCaT Cells in Response to LACCE by RNA-Seq

**Figure 5:**
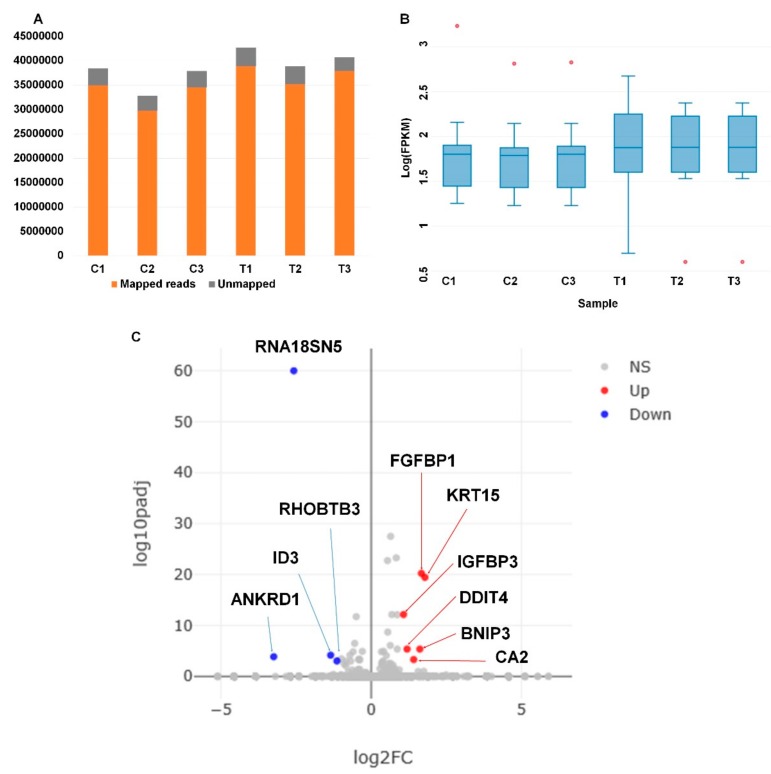
Mapping results, distribution of fragments per kilobase of transcript per million (FPKM) values, and visualization of differentially-expressed genes. (**A**) Portion of mapped (orange) and unmapped (gray) reads on human reference transcriptome. (**B**) Boxplot showing distribution of FPKM values in each library. (**C**) Volcano plot displaying distribution of log_10_ (padj) and log_2_ (FC) for all expressed genes. Padj and FC indicate adjusted *p*-value and fold change, respectively. Ten identified DEGs are indicated with blue (down-regulated genes, Down), red (up-regulated genes, Up), and gray (not significantly expressed genes, NS) dots.

Although the LACCE extract showed several positive effects as a cosmetic agent, it might be noteworthy to examine the change in human transcriptome in response to LACCE. For this, we used 1% LACCE extract instead of a lower concentration because we assumed that the possible effect with a low concentration of LACCE on keratinocyte cells might be small. The HaCaT cells were treated with 1% LACCE (treatment), whereas the control HaCaT cells were treated with sterile water. Three biological replicates were applied for RNA-Seq. The number of sequence reads ranged from 32,776,672 to 42,640,000 (Table 5). More than 90% of reads in all six samples were mapped on the human reference transcripts containing 159,998 transcripts (Figure 5A). For gene expression analysis, we calculated fragments per kilobase of transcript per million (FPKM) values. As shown in Figure 5B, the FPKM values from the three treated samples were higher than those from the three control samples. After removing redundant transcripts, only 11,290 transcripts of 68,158 transcripts were expressed in human keratinocyte cells (Table S1).

Although we used a higher concentration of LACCE (1%) that was 10 times higher than the normal LACCE (0.1%) used for cosmetics, the transcriptome of human keratinocyte cells did not significantly change in response to LACCE treatment, as shown in the volcano plot (Table S1 and Figure 5C). Based on the adjusted *p*-value of less than 0.001 and log*2* (fold change) of more than 1, we identified a total of 10 differentially-expressed genes (DEGs) upon LACCE (Table 6). Of the 10 DEGs, the four down-regulated genes were genes encoding an inhibitor of DNA-binding 3, ankyrin repeat domain 1, Rho-related BTB domain-containing 3, and 18S ribosomal N5. The six up-regulated genes were genes encoding carbonic anhydrase 2, insulin-like growth factor-binding protein 3, keratin 15, BCL2-interacting protein 3, fibroblast growth factor-binding protein 1, and DNA damage-inducible transcript 4.

### 3.8. Functional Roles of Up-Regulated Genes in Response to LACCE

The impact of LACCE on the keratinocyte transcriptome was milder than other stress treatments. To obtain a functional overview of up-regulated and down-regulated genes, we carried out GO enrichment analysis. However, we obtained no significant results for enriched GO terms due to a small of differentially-expressed genes. Therefore, we increased the number of differentially-expressed genes by applying an adjusted *p*-value of less than 0.05 and log_2_ (fold change) of more than 0.5. As a result, we identified 22 up-regulated genes and 13 down-regulated genes (Table S1). The GO enrichment analysis identified 36 enriched GO terms composed of 21 GO terms (biological process), two GO terms (molecular function), and 13 GO terms (cellular component) in up-regulated genes (Table S2). By contrast, only 12 enriched GO terms for the biological process were identified in 13 down-regulated genes (Table S2). 

**Figure 6:**
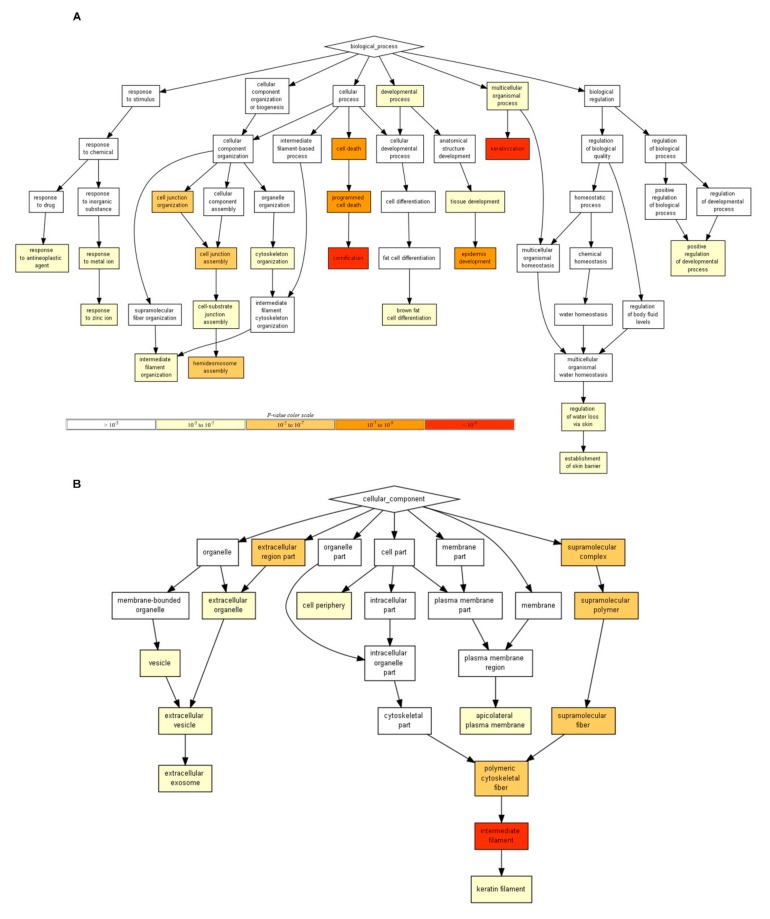
Hierarchical structure of identified enriched GO terms for up-regulated human genes in response to LACCE. Directed acyclic graphs (DAGs) visualize hierarchical structure of identified enriched GO terms for up-regulated genes upon LACCE treatment according to biological process (**A**) and cellular component (**B**). Each GO term is indicated by different box color based on *p*-value. Detailed information for identified GO terms can be found in Table S2.

According to the biological process, the genes involved in keratinization, cornification, programmed cell death, cell junction organization, the positive regulation of the developmental process, and the establishment of a skin barrier were highly up-regulated (Figure 6A). For the molecular function, the structural constituent of the cytoskeleton and structural molecule activity were highly expressed (Table S2). In the case of the cellular component, genes in the intermediate filament, extracellular exosome, and keratin filament were highly expressed (Figure 6B). Of 12 GO terms for 13 down-regulated genes, genes related to the response to zinc ion, regulation of ossification, and negative regulation of the biological process were frequently down-regulated.

## 4. Discussion

Cosmetics are classically defined as any prepared products that can be applied to the human body, including the face, skin, hair, mouth, and eyes, to change or strengthen the appearance of the human body [21]. In addition, cosmetics are used for cleansing, providing fragrance, and giving protection. Most cosmetics are composed of chemical compounds that can be derived from natural sources or synthetics [22]. 

In this study, we examined the possible effects of LACCE as a natural compound for cosmetics through diverse assays in vitro and in vivo. Several in vitro assay results showed the strong antioxidant activity of LACCE in response to UVB treatment. Interestingly, the effect of LACCE as an antioxidant was correlated with the concentration of LACCE. The antioxidant activity of LACCE (1%) was comparable to that of NAC or much higher than vitamin C. LACCE contains a higher amount of chlorogenic acid, 3,5-Dicaffeoylquinic acid, leontopodic acid B, and leontopodic acid A than normal edelweiss callus cultures, as shown in a previous study [4]. In particular, a previous study identifying two leontopodic acids from edelweiss demonstrated the functional role of LACCE as an antioxidant agent [23]. 

The expression of two inflammatory genes was suppressed by LACCE treatment, suggesting the possible role of LACCE in anti-inflammatory activity, as shown previously [7,24]. Interestingly, there was no significant difference in the anti-inflammatory effect among different LACCE concentrations, indicating that 1% LACCE might be sufficient for application as an anti-inflammatory agent in cosmetics. By contrast, the expressions of AQP3 and MMP2 required for moisturizing and wrinkling, respectively, were dramatically changed after treatment with different LACCE concentrations. 

A clinical test is the most important step for using plant extract as a cosmetic source. In this study, we examined the in vivo effects of LACCE with 21 volunteers. The application of LACCE on face and skin tissues showed significant increases in four different factors (improvements in periorbital wrinkles, skin elasticity, dermal density, and skin thickness) compared with the placebo. In particular, the constant use of LACCE improved the face and skin tissues significantly. Although edelweiss extracts are known as a source for cosmetics, this is the first report demonstrating the successful application of LACCE as a cosmetic material. 

To evaluate plant extracts as a cosmetic or medicinal source in vitro, the examination of the expression of marker genes is a popular experimental approach. However, the application of only selected genes for gene expression analysis has several limitations. The recent rapid development of next-generation sequencing facilitates gene expression analysis genome-wide. In this study, we used RNA-Seq to examine transcriptome-wide changes in human keratinocyte cells in response to LACCE. Our results showed that at least 16.56% of human genes were expressed in keratinocyte cells, indicating the tissue-specific expression of human genes. LACCE induced the expression of numerous genes; however, the global change in human transcriptome by LACCE was mild compared with other stress conditions. This result assures the safety of LACCE for application in human tissues. 

GO enrichment analysis revealed that up-regulated genes encoding Keratin 5 (KRT5), KRT19, KRT6A, KRT15, KRT14, KRT17, and junction plakoglobin (JUP) were involved in keratinization and cornification. Epidermal keratinocytes play an important role as a barrier against diverse environmental factors [25]. Specifically, terminal differentiated epidermal cells develop into dead keratinocytes by programmed cell death referred to as cornification, forming a strong epidermal barrier [26]. After cell death, the cornified skin layer provides many advantages to the face and tissues, including an increase in elasticity, stability, moisturizing, and mechanical resistance [25]. Of the identified KRT genes, the gene encoding Keratin15 (KRT15), a member of the keratin gene family, is known as a hair follicle stem cell marker that is highly expressed in skin tissues [27,28]. A recent study has suggested that KRT15 functions in epithelial regeneration against radiation and wound repair [29,30]. 

In addition to several genes encoding KRT proteins, genes encoding DDIT4, BNIP3, and IGFBP3 function in programmed cell death. For example, DNA damage-inducible transcript 4 (DDIT4) is highly expressed by different stresses and inhibits the mammalian target of rapamycin complex 1 (mTORC1) pathway associated with the treatment of cancer [31]. Previous studies have identified the DDIT4 gene, which was highly expressed by dexamethasone, which is a chemotherapeutic agent inducing autophagy in lymphocytes, suggesting its possible role in the regulation of cell growth, proliferation, and survival [32,33]. LACCE showed similar effects to dexamethasone as an anti-inflammatory agent. Bcl-2/adenovirus E1B 19-kDa-interacting protein (BNIP3) is a member of a pro-apoptosis protein family regulating cellular proliferation [34]. In addition, BNIP3 is involved in the protection of keratinocytes from UVB-induced apoptosis by up-regulating its gene expression [35]. Several studies have shown that insulin-like growth-factor-binding protein 3 (IGFBP-3) expression is related to cellular senescence [36,37]. The addition of IGFBP-3 induces or inhibits apoptosis depending on cell types [37]. For example, IGFBP-3 was up-regulated in the human papillomavirus-immortalized cervical cells, resulting in the enhancement of IGF-1-induced mitogenesis [37]. A recent study has shown the down-regulation of IGFBP-3 in senescent and H_2_O_2_-induced old cells compared with young cells, suggesting its possible role as an aging marker [36]. 

BNIP3, IGFBP3, stratifin (SFN), CA2, FGFBP1, KRT17, and JUP are involved in the positive regulation of the developmental process. Of them, carbonic anhydrases are ubiquitous enzymes present in prokaryotes and eukaryotes, including animals and plants [38]. In humans, carbonic anhydrase catalyzes the formation of carbonic acid from water and carbon dioxide (CO_2_), which are required for brain, kidney, and bone physiology [39]. A previous study demonstrated that CA4 and CA9 were up-regulated in a mouse during the wound hypoxic period by RNA-Seq [40]. In addition, the addition of recombinant CA9 enzyme promoted wound re-epithelialization [40]. Furthermore, a proteome study also revealed the involvement of CA2 protein in aging and neurodegeneration in a mouse [41]. Here, we demonstrated that LACCE promoted the regeneration of human keratinocyte cells by up-regulating CA2 expression. Fibroblast growth- factor-binding protein 1 (FGFBP1) is an extracellular secreted chaperone binding to FGFs and it modulates FGF signaling [42]. Several studies have shown that FGFBP1 is highly expressed during angiogenesis and plays important roles in skin carcinogenesis, inflammation, and wound healing [42,43,44]. Moreover, the cellular localization of up-regulated genes in the vesicle, extracellular exosome, intermediate filament, and keratin filament suggests that these proteins are a primary component of the cytoskeleton. 

In down-regulated genes, most genes are known to be induced by different stresses. For instance, two genes encoding metallothionein 2a (MT2A) and metallothionein 1e (MT1E) are induced under metal stress conditions, including zinc ion, copper ion, and cadmium ion, and oxidative stress, such as UVB [45,46]. Moreover, an inhibitor of DNA-binding 3 (ID3), a member of helix–loop–helix proteins, is an immediate–early gene in response to mitogenic signals and oxidative stress [47]. Ankyrin repeat domain 1 (ANKRD1), also known as cardiac ankyrin repeat protein (CARP), is a transcriptional cofactor that is up-regulated during wound healing and induces angiogenesis [48]. Both ID3 and ANKRD1 are involved in the negative regulation of the biological process. ID3 acts as a transcriptional regulator inhibiting stem cell differentiation and promoting cell cycle progression [47]. In addition, a recent study showed that the loss of ANKRD1 function in mice resulted in delayed wound healing, suggesting its role in wounding and tissue injury [49]. Furthermore, Rho-related BTB domain-containing 3 (RHOBTB3) functions to promote the proteasomal degradation of hypoxia-inducible factors (HIFs), which are the main regulators of adaptive responses to low oxygen [50]. In addition, interferon-induced transmembrane protein 1 (IFITM1) inhibits the entry of many viruses in the host cell [51]. Similarly, the gene encoding interferon alpha-inducible protein 6 (IFI6) is induced by interferon and regulates apoptosis and antiviral innate immunity [52,53]. The down-regulation of stress-responsive genes by LACCE treatment suggests that LACCE does not cause stress in human keratinocyte cells. 

## 5. Conclusions

Here, we evaluated LACCE as a natural cosmetic material through several in vitro and in vivo assays, suggesting its strong effects on the improvement of face and skin tissues. In particular, RNA-Seq-based transcriptome analysis revealed the molecular mechanism in human keratinocyte cells in response to LACCE. LACCE up-regulated the gene involved in keratinization and cornification, providing a skin barrier, which is promoted by genes required for programmed cell death. Many positive regulators of the developmental process were up-regulated, while diverse stress-induced genes were down-regulated by LACCE treatment. Taken together, our study demonstrated that LACCE is an agent for anti-aging cosmetics or cosmeceuticals.

## Figures and Tables

**Table 1:** Formulations to calculate the decreased rate and increased rate for clinical test.

Purpose	Formulation
The decreased rate	Rate of decrease (%) = (measurement prior to sample use − measurement after sample use)/measurement prior to sample use × 100
The increased rate	Rate of increase (%) = (measurement after product use − measurement before product use)/measurement before product use × 100

**Table 2:** Rates of decrease of roughness parameters of periorbital wrinkles (%).

			After 2 Weeks	After 4 Weeks
Rates of decrease (%)	LACCE	Ra	3.654	6.118
		Rq	3.583	6.189
	Placebo	Ra	1.330	2.490
		Rq	1.537	2.681

**Table 3:** Rates of increase in skin elasticity parameters (%).

			After 2 Weeks	After 4 Weeks
Rates of increase (%)	LACCE	R2	1.923	3.296
		R5	2.460	5.816
		R7	3.040	6.756
	Placebo	R2	1.081	1.621
		R5	1.113	2.895
		R7	1.689	3.378

**Table 4:** Rates of increase in dermal density and skin thickness (%).

			After 2 Weeks	After 4 Weeks
Rates of increase (%)	Dermal density	LACCE	3.062	5.466
		Placebo	2.249	3.118
	Skin thickness	LACCE	7.067	10.192
		Placebo	4.146	4.829

**Table 5:** Summary of raw sequence data and accession numbers.

Sample	Condition	Accession Number	Total Reads (bp)	Total Reads	Guanine-Cytosine GC(%)
C1	Control sample replicate 1	SRR9127735	3,875,300,108	38,369,308	50.05
C2	Control sample replicate 2	SRR9127736	3,310,443,872	32,776,672	49.9
C3	Control sample replicate 3	SRR9127737	3,820,715,264	37,828,864	49.94
T1	LACCE-treated sample replicate 1	SRR9127738	4,306,640,000	42,640,000	50.31
T2	LACCE-treated sample replicate 2	SRR9127733	3,919,963,924	38,811,524	50.53
T3	LACCE-treated sample replicate 3	SRR9127734	4,102,275,590	40,616,590	50.17

**Table 6:** Ten representative differentially-expressed genes upon LACCE treatment.

Gene Expression	Accession Number	Gene Function	Gene Symbol	log2 (Fold Change)	*p*-Value
Down-regulated	NM_002167.4	inhibitor of DNA binding 3, HLH protein	ID3	−1.352225574	1.33E-07
Down-regulated	NM_014391.2	ankyrin repeat domain 1	ANKRD1	−3.246186082	2.88E-07
Down-regulated	NM_014899.3	Rho related BTB domain containing 3	RHOBTB3	−1.143226842	2.39E-06
Down-regulated	NR_003286.4	RNA, 18S ribosomal N5	RNA18SN5	−2.579638571	3.86E-211
Up-regulated	NM_000067.2	carbonic anhydrase 2	CA2	1.410938268	1.2145E-06
Up-regulated	NM_000598.4	insulin like growth factor binding protein 3	IGFBP3	1.073197686	5.02E-16
Up-regulated	NM_002275.3	keratin 15	KRT15	1.785827844	1.96E-23
Up-regulated	NM_004052.3	BCL2 interacting protein 3	BNIP3	1.614937875	6.09E-09
Up-regulated	NM_005130.4	fibroblast growth factor binding protein 1	FGFBP1	1.668660016	2.60E-24
Up-regulated	NM_019058.3	DNA damage inducible transcript 4	DDIT4	1.192362381	5.33E-09

The 10 differentially-expressed genes upon LACCE treatment were identified based on adjusted *p*-value less than 0.001 and log_2_ (fold change) more than 1.

## Supplementary Materials

The following are available online at https://www.mdpi.com/2073-4425/11/2/230/s1: Table S1. Expression profiles of human keratocyte (HaCaT) cells in response to LACCE. Table S2. Identified enriched GO terms in up-regulated and down-regulated genes, respectively, in response to LACCE.
